# Combined metabolomic and genomic analyses reveal phage-specific and infection stage-specific alterations to marine *Roseobacter* metabolism

**DOI:** 10.1093/ismeco/ycaf047

**Published:** 2025-03-18

**Authors:** Min Jin, Lanlan Cai, Longfei Lu, Meishun Yu, Rui Zhang

**Affiliations:** State Key Laboratory Breeding Base of Marine Genetic Resource and Laboratory for Southern Marine Science and Engineering Guangdong Laboratory (Zhuhai), Third Institute of Oceanography, Ministry of Natural Resources, Xiamen 361000, China; Earth, Ocean and Atmospheric Sciences Thrust, The Hong Kong University of Science and Technology (Guangzhou), Guangzhou 510000, China; Fourth Institute of Oceanography, Ministry of Natural Resources, Beihai 536000, China; State Key Laboratory Breeding Base of Marine Genetic Resource and Laboratory for Southern Marine Science and Engineering Guangdong Laboratory (Zhuhai), Third Institute of Oceanography, Ministry of Natural Resources, Xiamen 361000, China; Archaeal Biology Center, Synthetic Biology Research Center, Shenzhen Key Laboratory of Marine Microbiome Engineering, Key Laboratory of Marine Microbiome Engineering of Guangdong Higher Education Institutes, Institute for Advanced Study, Shenzhen University, Shenzhen 518000, China

**Keywords:** *Roseobacter*, roseophage, metabolomic, auxiliary metabolic genes, metabolism

## Abstract

Phages can reshape the metabolic network of hosts to support specific requirements for replication during infection. However, metabolomic profiling of phage-elicited host global metabolic alterations and the linkage of phage-encoded auxiliary metabolic genes to these alterations are understudied. In this study, the dynamics of intracellular metabolites of *Dinoroseobacter shibae* DFL12, a member of marine environmentally and biogeochemically relevant *Roseobacter* clade, in response to four distinct lytic roseophage infections were investigated. Metabolomic profiling indicated that roseophage infections significantly altered host metabolism in a phage-specific manner. Pathway enrichment analyses showed that the central carbon pathway and DNA, amino acid, and coenzyme metabolism were commonly altered by roseophages, revealing a central role of these pathways in phage replication. Furthermore, clear infection stage-specific host responses were observed, corresponding to different metabolic demands of phage replication in the early and late infection stages. Interestingly, the content of host vitamin B_1_, which is the essential nutrient provided by *D. shibae* to its symbiotic microalgae, increased in the early infection stage for most roseophages, implying that phage infection may impact the symbiosis of *D. shibae* with microalgae. Finally, combined metabolomic and phage genomics analyses showed that roseophages adopt different strategies to expand the host pyrimidine pool (recycling or *de novo* synthesis of pyrimidine nucleotides), and this difference was likely related to variation in the GC content between phage and host genomes. Collectively, these results highlight the potential importance of phage-specific and infection stage–specific host metabolic reprogramming in marine phage–host interactions, bacteria–microalgae symbiosis, and biogeochemical cycles.

## Introduction

Viruses are the most abundant and ubiquitous biological entities on the planet [1, 2]. They influence host communities and biogeochemical cycles significantly since ~10^28^ viral infections are estimated to occur in the ocean each day, killing 20%–40% of marine prokaryotes daily and releasing up to 10^9^ tons of carbon from cells [1, 2]. Evidence that the physiology and metabolism of infected cells (virocells) are distinct from those of their uninfected counterparts is accumulating, implying that biogeochemical models need to consider these two states separately [3–6]. Because most viruses lack their own metabolic machinery, they augment key steps in host metabolism to fuel viral replication during infection. To reprogram host metabolic processes, many viral genomes have acquired host-derived genes with metabolic functions, termed “auxiliary metabolic genes” (AMGs) [7]. Our knowledge of the number and functional diversity of AMGs has increased rapidly over the past decade owing to the increased availability of metagenomics data [8, 9]. AMGs found in marine phages include genes involved in photosynthesis, iron–sulfur clusters, phosphate, sulfur, and nitrogen metabolism, carbon metabolism, DNA metabolism, fatty acid metabolism, and other metabolic pathways [5–7].

In contrast to the increasing knowledge concerning the potential of phages to take over and regulate host metabolism based on genetic analyses, phage-elicited alterations in host global metabolism and the linkage of AMGs to these alterations are severely understudied. Ankrah *et al*. used metabolomic to investigate the influence of phage infection on the global metabolism of *Sulfitobacter*, revealing increases in the intracellular concentrations of 75% detected metabolites at the end of the phage infection cycle [10]. Their results also showed that small, labile nutrients in the lysate were utilized by surviving cells. Metabolomic profiling of *Geobacillus* sp. E263, a thermophilic bacterium isolated from a deep-sea hydrothermal vent, showed that various metabolites (tryptophol, adenine, and hydroxybenzyl alcohol) were significantly elevated upon phage infection, suggesting that these metabolites have roles in phage–host interactions in the extreme environments [11]. Transcriptomic and metabolomic profiling of phage–host interactions between phage PaP1 and *Pseudomonas aeruginosa* revealed the downregulation of the choline–glycine betaine pathway [12]. A high-coverage metabolomic analysis of multiple phage infections revealed phage-specific alterations to *P. aeruginosa* physiology during infection, emphasizing the potential importance of the “phage diversity” parameter when studying metabolic interactions in complex communities [13]. To our knowledge, the study by De Smet *et al*. was the only report to date to investigate in-depth the linkage between phage AMGs and metabolic reprogramming based on metabolomic profiling, where they found that phage-encoded AMGs reprogram the host metabolism in phage-specific ways [13]. Currently, little is known about the impact of phage infection on global host metabolomes beyond these limited isolated model systems. Since exploring how metabolic processes differ between infected and uninfected cells is crucial for estimating the impacts of phage infection on ecosystems and biogeochemical cycles [5], more work is needed to determine the extent to which these limited findings are shared across diverse phage–host systems.

*Roseobacter* are abundant, ubiquitous, and diverse in the ocean. Members of this alphaproteobacterial lineage account for up to 25% of marine microbial communities, especially in coastal regions and open oceans [14]. *Roseobacter* spp. play active roles in the global carbon and sulfur cycle and the climate since they are capable of aerobic anoxygenic photosynthesis, carbon monoxide oxidation, and sulfur compound transformation [15, 16]. *Roseobacter* species produce various secondary metabolites [17] and degrade aromatic materials [18]. *Dinoroseobacter shibae* DFL12, a member of the *Roseobacter* clade, was first isolated from *Prorocentrum lima*, a benthic toxic dinoflagellate [19, 20]. The genome sequence of *D. shibae* DFL12 revealed traits that are presumably highly adaptive in the habitat of bloom-forming algae [21]. For example, *D. shibae* DFL12 is able to synthesize the vitamins B_1_ and B_12_ for which its algal host is auxotrophic. More than 50 bacteriophages that infect multiple *Roseobacter* lineages have been isolated and sequenced. These roseophages exhibit diverse morphologies, nucleic acid types, and genomic features [22, 23]. Interactions between roseophages and *Roseobacter* have been characterized extensively by genomic, transcriptomic, and proteomic analyses [24–26]. However, little is known about the effects of roseophage infection on global *Roseobacter* metabolic processes [10]. In view of the environmental and biogeochemical relevance of the *Roseobacter* clade, as well as the central roles of viruses in shaping host metabolism and mediating biogeochemical cycles, exploring the influences of roseophage infection on global *Roseobacter* metabolism is essential. Additionally, although >180 AMGs have been identified in the genomes of roseophages [23], the linkage of these AMGs to roseophage-elicited host metabolic alterations remains unknown. Therefore, in order to explore roseophage-elicited alteration in *Roseobacter* metabolism and the possible linkage of roseophage-encoded AMGs to these alterations, we performed high-coverage metabolomic profiling of *D. shibae* DFL12 during the infection of the four distinct roseophages in this study.

## Materials and methods

### *Dinoroseobacter shibae* strain and phage isolates

*Dinoroseobacter shibae* strain DFL12 was originally isolated by plating washed single cells of cultivated *P. lima* onto an agar surface [19, 20]. The optimum doubling time of DFL12 is 0.12 h [19]. The complete genome sequence of DFL12 has been reported [21]. Four lytic phages infecting DFL12 were selected to represent a diverse set of *Roseobacter* bacteriophages, i.e., vB_DshP-R2C (R2C), vB_DshS-R4C (R4C), vB_DshP-R7L (R7L), and vB_DshS-R26L (R26L). The genomes of these *Roseobacter* bacteriophages have been sequenced, and the main characteristics of the genomes and infection cycles are listed in Table S1. All the studied roseophages are lytic viruses that lead to the lysis of the hosts and the release of phage progenies [22, 27–29]. Phage stocks were prepared based on PEG8000 precipitation and cesium chloride (CsCl) gradient ultracentrifugation as described previously [22] and stored in SM (Salt Mg) buffer (50 mM Tris–HCl pH 7.5, 0.1 M NaCl, 8 mM MgSO_4_) at 4°C. Briefly, the phage was propagated in DFL12 and harvested in supernatant by centrifugation at 10 000×*g* for 10 min after complete bacterial lysis. The phage suspension was then filtered through a 0.2-μm membrane and was precipitated with 1 M NaCl and PEG8000 (10% w/v) overnight at 4°C. After centrifugation at 10 000×*g* for 60 min, the phage particles from the PEG pellet were resuspended in SM buffer and were then purified with CsCl gradient centrifugation (200 000×*g*, 4°C, 24 h). The phage bands were collected and dialyzed against SM buffer at 4°C.

### Clustering of *D. shibae* phages with all RefSeq phage genomes

The clustering of phages was performed by applying vConTACT2 using ProkaryoticViralRefSeq201-Merged as the reference database [30]. In brief, for each phage genome, open reading frames (ORFs) were called using Prodigal v2.6.3, and the predicted protein sequences were used to compute the gene-sharing network using vConTACT2. Diamond was used for protein–protein similarity analyses, with parameters set to default. The clustering results were visualized using Cytoscape 3.8.2 [31].

### Killing curve analysis of *D. shibae* phages

*Dinoroseobacter shibae* DFL12 was cultured in artificial seawater supplemented with 1 g/l glucose and 1 g/l sodium acetate at 37°C with shaking. When the OD_600_ reached 0.35, each phage was added to a bacterial culture, and the growth of host cells was monitored by recording OD_600_ values. To monitor host intracellular metabolites during all phases of infection, samples were collected at 0.5, 1, and 2 h post–phage infection (p.i.) for all four phages. Uninfected host cells were also collected at the same time points in parallel as controls. For each phage infection and uninfected control, four biological replicates were sampled for each time point.

### AMG identification and annotation

AMGs were identified from phage genomes using VIBRANT v1.2.0105 [32] with “virome” parameters. Then, the putative AMGs were further annotated using the NCBI blast tool (https://blast.ncbi.nlm.nih.gov/Blast.cgi) against the NR database by “blastp.”

### Metabolite extraction and liquid chromatograph tandem mass spectrometer analysis

Host cells were harvested by centrifugation at 5000×*g* at room temperature for 4 min to remove extracellular compounds. To terminate bacterial metabolism, the cell pellet was washed twice with 0.9% sodium chloride solution and resuspended in extraction solution (50% acetonitrile with 0.1 M formic acid). Then, the resuspended solution was subjected to vigorous shaking at 4°C for 15 min to extract intracellular metabolites. The supernatant containing the extracted metabolites was separated from the cell debris by centrifugation at 15 000×*g* at 4°C for 5 min. To accomplish the efficient extraction of intracellular metabolites from the bacteria, the resulting cell debris was repeatedly extracted with the extraction solution thrice, and the supernatants containing the extracted metabolites were combined, followed by the addition of L-2-chlorophenylalanine as the internal standard for quality control. The metabolite extracts were lyophilized and resuspended in 200 μl of 50% acetonitrile for untargeted high-coverage liquid chromatography tandem mass spectrometry (LC–MS/MS).

LC–MS/MS analyses were performed using an ultra–high-performance liquid chromatography (UHPLC) system (1290; Agilent Technologies) with a UHPLC BEH Amide column (1.7 μm 2.1 × 100 mm; Waters) coupled to the TripleTOF 6600 (Q-TOF; AB Sciex). LC–MS/MS analyses, one each in positive and negative ion modes, were performed for each biological replicate. The mobile phase consisted of 25 mM NH_4_OAc and 25 mM NH_4_OH in HPLC-grade water (pH = 9.75) (solvent A) and HPLC-grade acetonitrile (solvent B). The gradient elution profile was as follows: 0 min, 5% solvent A, 95% solvent B; 7 min, 35% solvent A, 65% solvent B; 9 min, 60% solvent A, 40% solvent B; 9.1 min, 5% solvent A, 95% solvent B; 12 min, 5% solvent A, 95% solvent B. The flow rate was 500 μl/min. The TripleTOF mass spectrometer was operated in data-dependent acquisition mode to acquire MS/MS spectra within the control of the acquisition software (Analyst TF v1.7; AB Sciex). In each cycle, 12 precursor ions with intensity >100 were chosen for fragmentation at a collision energy of 30 V, and MS/MS spectra were acquired at a rate of 15 events per 50 ms. Electrospray ionization parameters were set as follows: nebulizing gas, 60 psi; auxiliary gas, 60 psi; source temperature, 650°C; spray voltage, 5000 V for positive ion mode or 4000 V for negative ion mode.

### Metabolomic data analysis

Raw mass spectrometry data files (.d) were converted to the mzXML format using ProteoWizard [33] and processed using the R package XCMS [34] to generate a data matrix consisting of the retention time, mass to charge ratio (*m*/*z*), and peak intensity. Each peak intensity was normalized according to the total ion current. The normalized data matrix was input into SIMCA (V14.1; MKS Data Analytics Solutions, Umea, Sweden) for a supervised orthogonal projection to latent structures–discriminate analysis (OPLS-DA). OPLS-DA could reveal a high level of group separation as well as variables responsible for group separation [35]. A permutation plot was generated to estimate the robustness and predictive ability of the OPLS-DA model. The high values of the *Q*^2^ intercept indicated the robustness of OPLS-DA models, thus showing a low risk of overfitting. Based on the OPLS-DA, variable importance in projection (VIP), reflecting the contribution of variables to group separation, was computed for each peak. To identify significantly altered metabolite peaks associated with each phage infection, the intensity for the same peak was compared between the phage-infected and uninfected host cells at each time point. Phage-infected and uninfected host groups were compared using Student’s *t*-tests. Metabolite peaks were considered significantly changed when *P* ≤.05 and VIP ≥1, and results were visualized by a volcano plot. An in-house MS/MS database was used for metabolite peak identification. To ensure accurate metabolite identification, only metabolite peaks with MS/MS identification score >0.6 were retained for further metabonomics analyses. Hierarchical clustering of metabolites was performed using the R package pheatmap v1.0.12 [36]. For a metabolic pathway enrichment analysis, MetaboAnalyst v5.0 [37] was utilized based on the Kyoto Encyclopedia of Genes and Genomes (KEGG) database [38], with the enrichment method of hypergeometric test and topology measure method of relative-betweenness centrality.

## Results and discussion

### Diversity of selected roseophages

**Figure 1:**
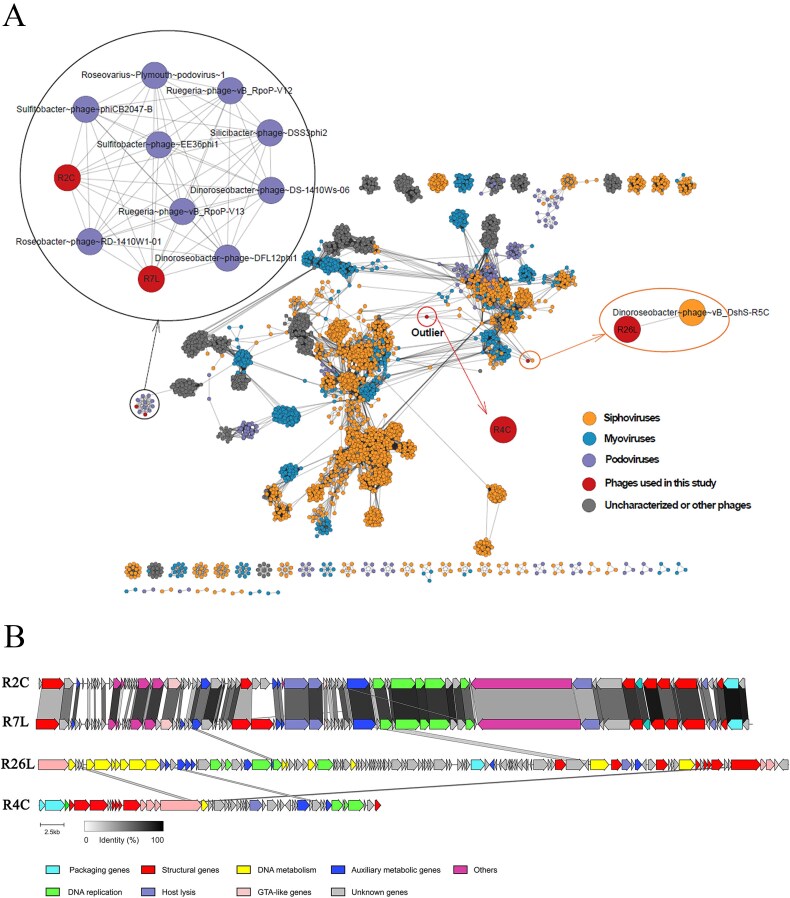
Genomic diversity of the four *Dinoroseobacter shibae* phages*.* (A) Clustering of the four roseophage with all RefSeq phage genomes (v201). Clustering was based on 4534 phage genomes belonging to siphoviruses, myoviruses, podoviruses, or uncharacterized and other phages. Edges indicate the significant pairwise similarity between viruses (nodes) in terms of shared protein contents. The affiliated phage groups for four studied roseophages are shown in circles and named in bold. (B) Genome comparison of the four roseophages. Genes are colored according to their annotated functional modules. Arrows represent the direction of gene transcription, and the gradient scale indicates the sequence identity range between phage genes.

*Dinoroseobacter shibae* strain DFL12, a type strain from the *Roseobacter* clade, was selected as the bacterial host system owing to its extensive genetic and phenotypic resources [19, 21, 39, 40], metabolic versatility [14, 21], novel acylated homoserine lactone (AHL) compounds [41], and symbiosis with microalgae [42, 43]. To investigate phage-specific effects on host metabolic processes, four roseophages (R2C, R4C, R7L, and R26L) that infect DFL12 were selected as diverse representatives within the broad roseophage universe. Transmission electron microscopy showed that R2C and R7L are podoviruses with a short non-contractile tail, while R4C and R26L are siphoviruses with a long flexible tail (Fig. S1). To date, >50 roseophages that infect multiple clades of *Roseobacter* have been isolated and sequenced. The majority of reported roseophages belong to either podoviruses or siphoviruses [22, 23], which are both represented by our roseophage selections. In a comparative genomics analysis, 32 sequenced roseophages clustered into at least 8 distinct groups, with few genes shared between each group (up to 8%), indicating the high diversity of roseophage genomes [44]. The genomes of the four roseophages in this study have been sequenced, and the main genomic characteristics are listed in Table S1. To illustrate the genomic diversity of the selected roseophages, a phage gene-sharing network was constructed based on protein sequence similarity (Fig. 1A). The four selected roseophages represented different clusters within this network. R2C and R7L were clustered within the N4-like roseophage group, while R26L clustered with roseophage R5C [45], which also infects strain DFL12. By contrast, R4C did not cluster with any known roseophages or with any known bacteriophages in the RefSeq database (Fig. 1A). Similarly, the comparative genomic analysis showed that R2C and R7L were highly similar in terms of genome size, GC content, genomic arrangement, and gene similarity (Fig. 1B), consistent with the previous observation that all N4-like roseophages isolated from different geographic locations and infecting different hosts share a high level of genomic conservation [46]. Nevertheless, R2C and R7L remain different phages, as their genomes share an average nucleotide identity of 79.5%. Furthermore, both the killing curve analysis (Fig. S1b) and infection cycle inferred from the one-step growth curve analysis (Table S1) showed that the selected roseophages have diverse infection phenotypes. Collectively, the four studied phages captured substantial diversity with respect to genomic characteristics and infection phenotypes.

### Roseophage infection significantly affects host metabolism

**Figure 2:**
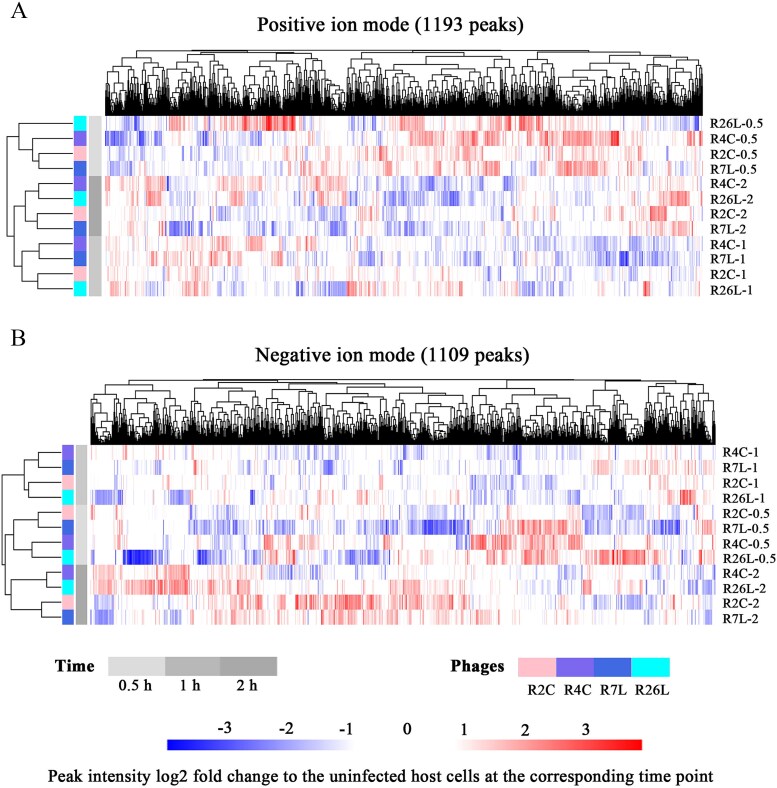
Hierarchical clustering based on the fold change of mean peak intensities for intracellular metabolites of phage-infected cells relative to control populations. Peaks (columns) measured in positive (A) or negative ion mode (B) are shown for the different samples (rows). Fold changes are log2-transformed. Hierarchical trees indicating the relation between different samples or different peaks are shown on the left or top, respectively.

**Figure 3:**
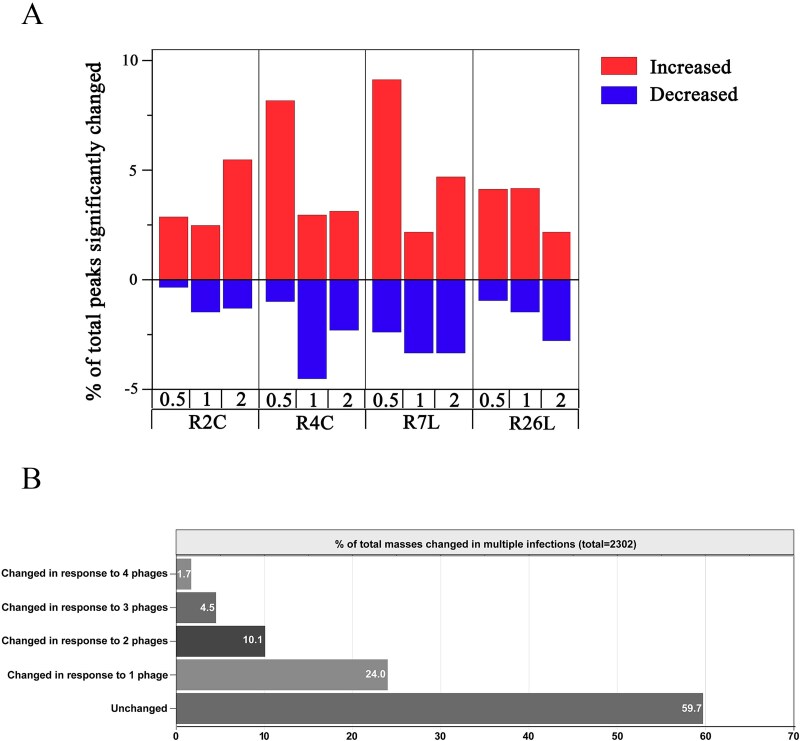
(A) Percentage of total peaks significantly changed (*P* ≤ .05 and VIP ≥ 1) between phage-infected and control populations during the course of infection of *Dinoroseobacter shibae* DFL 12 with four different phages. (B) The percentage of total peaks significantly changed (*P* ≤ .05 and VIP ≥ 1) and shared among multiple phage infections. Significantly changed intracellular metabolite peaks were obtained by comparisons of the peak intensity in the phage-infecting host cells with that in the uninfected host cells at the corresponding time point. The metabolite peak was considered significantly changed in a phage only if it was significantly changed at least one time point during the phage infection.

To explore the dynamic response of DFL12 metabolism to infection by different roseophages, host intracellular metabolites were sampled with four biological replicates at different timepoints p.i. (0.5, 1.0, and 2.0 h, Fig. S1b). We chose these time points because they correspond with different stages of infection cycles (early and late infection stages) and the post-latent period of these roseophages to the greatest extent. According to the latent period (Table S1), the sampling time point of 0.5 h p.i., 1 h p.i., and 2 h p.i. corresponded with the early infection stage, late infection stage, and post-latent period, respectively, for the three roseophages (R2C, R4C, and R7L). The latent period of R26L is 3.5 h (Table S1), which differs from those of three other roseophages, causing difficulty in choosing sampling timepoints for synchronized stages across roseophages. Nevertheless, the R26L infection was sampled at the same timepoints (0.5 h p.i., 1 h p.i., and 2 h p.i.), as it is easier to compare the results of different roseophages at the same sampling timepoints. The extracted metabolites were then analyzed by untargeted UHPLC coupled TripleTOF mass spectrometry in both positive and negative ion modes, as this method enables more comprehensive metabolome coverage than using a single ion mode [47]. Twelve repeated LC–MS/MS analyses of the same quality control sample were performed, showing a good correlation (>0.97) (Fig. S2), indicating the outstanding stability and accuracy of our LC–MS/MS measurements. A total of 2302 peaks were identified from 84 individual LC–MS/MS analyses (each in both positive and negative ion modes), of which 1193 and 1109 peaks were acquired from positive (Table S2) and negative ion (Table S3) modes, respectively. To compare the metabolite dynamics during phage infection, peak intensity fold changes were determined for each phage infection relative to the uninfected control at the corresponding time point (e.g., R2C-0.5 h versus Control-0.5 h). Hierarchical clustering based on the peak intensity fold change values showed that roseophage infections altered host metabolism significantly, as a large proportion of host metabolite peaks increased or decreased in relative abundance (≥1.5-fold change) during the course of phage infections (Fig. 2, Table S4, and Table S5). Compared with the uninfected control, up to 11.5% of the detected metabolite peaks were significantly changed (VIE > 1 and *P* < .05, Tables S3 and S4 and Fig. S3) after phage infection at a single time point, with the most changes observed in the R7L-0.5 h sample (9.1% of peaks increased significantly and 2.4% decreased) (Fig. 3A and Table S6). During the course of the four roseophage infections, 40.3% of the detected metabolite peaks changed significantly (VIE > 1 and *P* < .05) in response to at least one phage (Fig. 3B). Consistently, OPLS-DA also showed a clear separation of phage-infected host metabolomes from uninfected host metabolomes (Fig. S4). These results were consistent with previous findings for the infection of *Sulfitobacter* [10] and *Pseudomonas* phages [13], suggesting that host metabolism is significantly altered by phage infection in different host systems. The lytic infection of phage Φ2047B caused alterations in ~25% of the 82 measured metabolites in *Sulfitobacter* sp. 2047 after a single infection cycle [10], while 56% of the 518 detected metabolites changed significantly in *P. aeruginosa* strain PAO1 during multiple phage infections [13]. However, it should be noted that the methods used for LC–MS/MS analysis, metabolite identifications, the statistical tests, and thresholds used for calling a significantly changed metabolite vary between this study and these two studies, which may cause bias during comparisons.

### Distinct host responses to different roseophage infection

Although roseophage infection affected host metabolism significantly, only 1.7% of the detected metabolite peaks changed significantly upon infection with all four phages (Figs 3B and S5), indicating a lack of a universal host response to different roseophage infections. By contrast, 24% of the detected peaks changed significantly unique to a single phage. This result is in line with previous observation in the *P. aeruginosa* system, where only 2.4% of the detected metabolites changed significantly in response to six *Pseudomonas* phages simultaneously [13]. The four studied roseophages displayed distinct temporal patterns with respect to metabolomic responses during the course of phage infections, further confirming the absence of a universal host response (Fig. 3A and Table S6). There is growing evidence that host responses to phage infections are highly specific to the selected phage–host pair. This specificity is evident in the highly variable host transcriptional responses to infection [5]. For example, transcriptome and proteome studies of *Pseudoalteromonas* showed that the siphovirus PSA-HS2 alters host nucleotide and amino acid metabolism, while the podovirus PSA-HP1 represses energy-consuming metabolic pathways, including motility and translation [48]. Besides, a transcriptomic study on interactions between the T4-like cyanophage Syn9 and three phylogenetically, ecologically, and genomically distinct marine *Synechococcus* strains showed that the hosts mounted a gene-specific response to infection even though they were infected by the same phage [49]. However, whether phages can trigger different host responses is likely associated with the genomic and physiological characteristics of the phages [13, 49].

The heatmap of peak fold changes relative to the uninfected control showed that most of the differential peaks did not increase or decrease in relative abundance unilaterally during the course of phage infection (Fig. 2). For example, host *N*-acetyl-l-glutamate remained stable at 0.5 h p.i., decreased at 1 h p.i., and finally increased at 2 h p.i. in response to the infections with R2C, R7L, and R26L (Fig. S6). The temporal fluctuations in metabolite contents in the *D. shibae* system disagreed with the observed pattern in the phage Φ2047B–*Sulfitobacter* system, where phage infection led to a progressive and general increase in concentrations of nearly all measured compounds [10]. This result highlights the remarkable variation in metabolic responses to phage infections among members of the *Roseobacter* lineage. However, it should be noted that this variation may be caused by the differences in methods for metabonomics generation and data analysis between different studies.

### Roseophages reprogram host metabolisms in an infection stage–dependent manner

**Figure 4:**
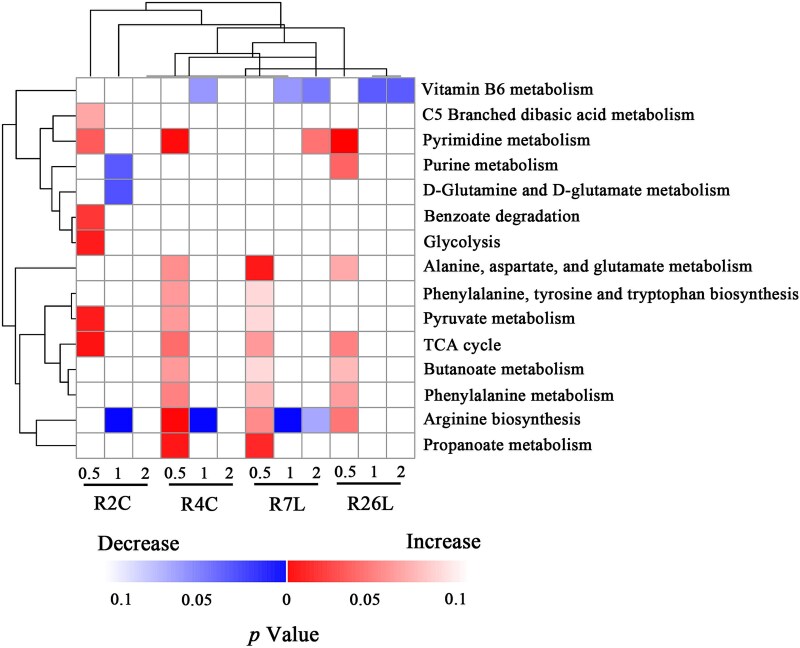
Metabolic pathway enrichment analysis for four different phages during the course of infection. Significantly decreased and increased pathways are shown. The color scale indicates the *P*-values.

An in-house MS/MS database was applied in metabolite peak identification. To ensure accurate metabolite identification, only metabolite peaks with an MS/MS identification score of >0.6 were retained for functional enrichment analyses (Table S2 and Table S3). Pathway enrichment analysis was performed to identify upregulated or downregulated pathways enriched for all the significantly increased or decreased metabolites, respectively. As shown in Fig. 4, notable temporal variations in enriched pathways were found for each roseophage, consistent with the temporal variation observed in the content of metabolite peaks (Fig. 2). Most of the enriched pathways could be linked to specific requirements of phages at different stages of infection. In the early infection stage, most of the enriched metabolic pathways were those enriched for increased metabolites (13 out of 14 enriched pathways, 92.9%), including TCA cycle, amino acid metabolic pathway, and pyrimidine pathway (Fig. 4). First, the TCA cycle, which provides energy as well as metabolic precursors for the biosynthesis of amino acids and fatty acids, was enriched for significantly increased metabolites in the early infection stage for all the four analyzed roseophages. Interestingly, glycolysis, another central carbon metabolism pathway, was only enriched for increased metabolites in the early infection stage of R2C. Glycolysis drives the production of energy in the form of ATP, NADH, and NADPH and supplies the carbon necessary for the synthesis of numerous core biomolecules, including nucleotides, lipids, amino acids, and carbohydrates [50]. The activation of the TCA cycle and glycolysis have been revealed for a wide variety of viruses, especially for eukaryotic viruses [51]. Second, several amino acid metabolic pathways were upregulated for most roseophages in the early stage of infection, including alanine, aspartate, and glutamate metabolism, arginine biosynthesis, phenylalanine metabolism, and phenylalanine, tyrosine, and tryptophan biosynthesis. This upregulation can be explained by an increase in the synthesis of amino acids or the induction of protein catabolic pathways, which was evidenced by the abundant dipeptides in the significantly increased metabolite set shared among all or three phage infections (Table S7 and Fig. S6). Dipeptides are intermediates of protein digestion or protein catabolism. Third, the pyrimidine pathway, which produces pyrimidine nucleotides required for phage DNA replication, was enriched for significantly increased metabolites for R2C, R4C, and R26L in early infections. In contrast, purine metabolism was enriched for significantly increased metabolites in response to R26L in early infection and even enriched for significantly decreased metabolites by R2C in late infection. The dissimilarity of host responses between purine and pyrimidine pathways was also found in *P. aeruginosa* systems; De Smet *et al*. suggest that there is a difference in the consumption of pyrimidine versus purine nucleotides during phage infection [13]. Besides, we speculate that the dissimilarity of host responses between purine and pyrimidine pathways may also be associated with the AMG set carried by phages since some of the roseophages studied here encode abundant AMGs involved in pyrimidine metabolism but no AMGs involved in purine metabolism. Collectively, these results indicated that roseophages redirect host metabolic pathways to generate essential building blocks and energy to fuel phage DNA and protein synthesis in the early stage.

In the late infection stage and post-latent period, most host metabolic pathways remained stable, except that arginine biosynthesis and vitamin B_6_ metabolism were enriched for significantly decreased metabolites for most analyzed roseophages (Fig. 4). The genome of *D. shibae* DFL12 harbors the hypothetical arginine deiminase pathway, which allows strain DFL12 to carry out a fermentation process for survival known from *Bacillus* and *Pseudomonas* species [21]. Thus, the decrease in l-citrulline in late infection stage might be due to host arginine deiminase survival fermentation in order to sustain active energy metabolism. Vitamin B_6_ is an essential coenzyme of many diverse enzymes involved in amino acid metabolism in all cells [52]. The decrease in vitamin B_6_ may lead to a global reduction in host amino acid metabolism in the late infection stage and post-latent period. However, the exact role of vitamin B_6_ in the metabolic interaction between roseophages and hosts needs to be further elaborated. Taken together, our metabolomic results indicate that roseophages reprogram host metabolisms in an infection stage-dependent manner, which is likely to support varying metabolic demand at different stages of phage infection [4, 51].

It is well recognized that metabolic reprogramming of infected cells and viral lysis alter nutrient cycling and biogeochemical cycles in the oceans, although the net impacts remain unknown [5]. The biogeochemical influence of viruses begins during viral infection when they remodel host metabolisms [4] and continues even after host lysis since the cellular debris is released into the surrounding environment where they are utilized by the wider microbial community that catalyzes biogeochemical transformations [5]. In this context, several models have been constructed to evaluate the biogeochemical influence of viral activity and stress the need to incorporate viruses into biogeochemical models [53, 54]. However, our study shows that phages reprogram host metabolism in the phage-specific and infection stage-specific manners, implying that not only the occurrence of phage infection but also the diversity of phages and infection stage will impact the net metabolic effect in complex microbial communities. These factors should be considered when exploring the global ecological and biogeochemical roles of phages in complex systems in nature.

### Pathways commonly targeted by roseophages

**Figure 5:**
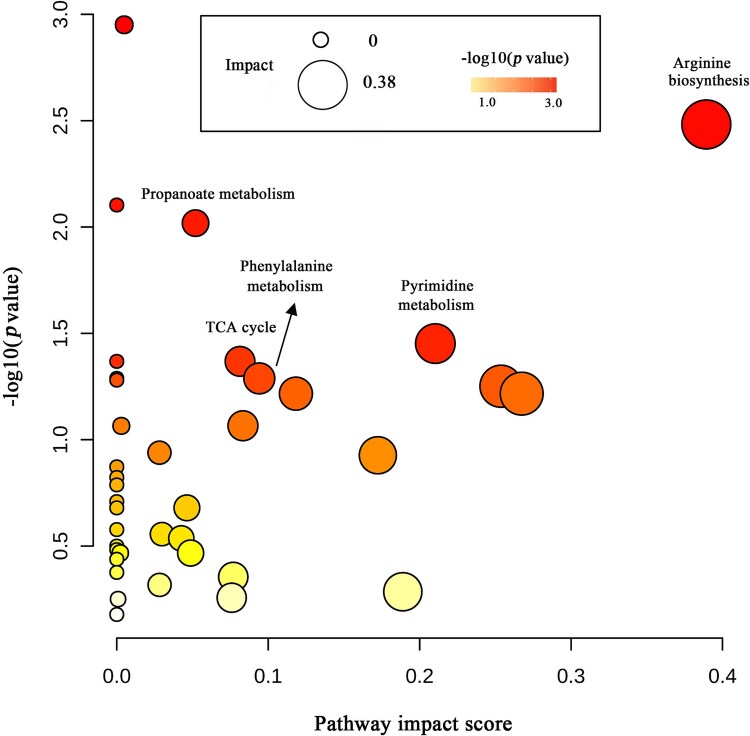
Pathway analysis of significantly changed intracellular metabolites shared in all or three phage infections. The color and the size of bubbles are proportional to the –log10 (*P*-value) and pathway impact score, respectively. Only enriched pathways with pathway impact ≥0.05 and *P* <0.05 were annotated.

To identify the pathways commonly targeted by DFL12 phages, a pathway enrichment analysis was performed on the significantly changed metabolites shared by at least three roseophages. One hundred and forty-three metabolite peaks significantly changed during infections with at least three phages, accounting for only 6.2% of the total detected peaks (Fig. 3B). Among 143 shared peaks, 42 peaks were identified with MS/MS matches (Table S7 and Fig. S6), and those were mainly involved in the TCA cycle and DNA, amino acid, and coenzyme metabolism. Indeed, the pathway enrichment analysis showed that roseophages commonly targeted host pyrimidine metabolism, arginine biosynthesis, phenylalanine metabolism, TCA cycle, and propanoate metabolism (Fig. 5). Notably, some of the commonly changed metabolites by four roseophages have potentially important roles in phage propagation and/or are ecologically relevant. For example, hypoxanthine, a purine derivative, has been shown to inhibit DNA replication by DNA polymerase of *P. aeruginosa* phage PaP1 [55]. The content of hypoxanthine increased in the early infection stage and was stable or decreased in the late infection stage for most roseophages (Table S7 and Fig. S6). The role of hypoxanthine in DFL12–roseophage interactions is unknown, but we speculate that it may participate in the host defense response against roseophage infection. l-Glutamate, *N*-acetyl-l-glutamate, adenosine, UDP, thymidine, and acetyl-CoA are components of central C and N metabolism. These metabolites released from lysed cells during viral lysis could be readily recycled by the surrounding microbial community to support their own growth [13]. Thus, the roseophage-induced changes in the content of these metabolites likely have ecological implications for the surrounding microbial community. A follow-up experiment exploring the extracellular metabolites in the viral lysates is needed in the future to verify this hypothesis.

### Potential linkages of phage-specific metabolic responses with predicted AMGs

In contrast to the increasing knowledge concerning the number and functional diversity of AMGs in the marine environment, as inferred from metagenomic data, experimental studies investigating the physiological and metabolic imprint of AMGs in hosts upon phage infections are scarce [4, 5]. The cost of maintaining additional genetic material is presumably high for small phage genomes, indicating the potentially important roles of AMGs in phage infection and replication [6]. To link phage-specific metabolic responses with phage AMGs, we identified abundant AMGs from all four available roseophage genomes, and most of them were predicted to participate in nucleotide metabolism (Table 1). The abundance of AMGs involved in nucleotide metabolism likely explains the notable impact on host purine and pyrimidine metabolism by most roseophages (Fig. 4), consistent with the central need for nucleotides in phage genome replication. Previous comparative metagenomics analysis of enriched AMGs from the Global Ocean Survey showed that the most enriched metabolic pathways in marine viral metagenomes are involved in nucleotide biosynthesis, and AMGs expanded the host nucleotide pool through two mechanisms, including the degradation and recycling of host nucleotides and directing the host metabolism to provide substrates for *de novo* synthesis [13, 56].

**Table 1:** Predicted tRNAs, nuclease, and AMGs present in the genome of *Dinoroseobacter shibae* phages used in this study.

Phage	tRNA	Predicted nuclease and AMGs in phage genome					
		ORF	Predicted function	Pathway	Top hit against NCBI NR database	Coverage | identity	Blast E-value
R7L	Ile, Pro	gp4	Nucleoside 2-deoxyribosyltransferase^*^	Nucleotide	Nucleoside 2-deoxyribosyltransferase [YP_009874076.1]	89% | 88.07%	7e-67
		gp25	HNH endonuclease	Nucleotide	HNH endonuclease [YP_009874013.1]	97% | 90.73%	0
		gp29	dCMP deaminase^*^	Nucleotide	Deoxycytidylate deaminase [YP_009874016.1]	98% | 78.72%	5e-79
		gp32	Thymidylate synthase^*^	Nucleotide	Thymidylate synthase [YP_002898958.1]	94% | 71.53%	8e-147
		gp42	Nucleotide pyrophosphohydrolase^*^	Nucleotide	Nucleotide pyrophosphohydrolase [YP_009880443.1]	95% | 78.49%	3e-95
		gp43	Thioredoxin^*^	Oxidative stress response/nucleotide	Thioredoxin domain [YP_009043724.1]	100% | 58.10%	4e-39
		gp46	HNH endonuclease	Nucleotide	Endonuclease [YP_009874034.1]	96% | 89.90%	1e-60
		gp55	Ribonucleotide reductase^*^	Nucleotide/glutathione	Hypothetical host-like ribonucleoside diphosphate reductase [CBW47044.1]	100% | 85.28%	0
		gp60	Exonuclease	Nucleotide	Exonuclease [YP_009874048.1]	100% | 91.54%	0
R2C	Ile, Pro	gp21	HNH endonuclease	Nucleotide	HNH endonuclease [YP_009043742.1]	92% | 99.76%	0
		gp23	dCTP deaminase^*^	Nucleotide	dCTP deaminase [YP_009043740.1]	100% | 100.00%	4e-116
		gp27	Thymidylate synthase^*^	Nucleotide	Putative thymidylate synthase [YP_009043736.1]	100% | 99.39%	0
		gp39	Thioredoxin^*^	Oxidative stress response/nucleotide	Nucleotide thioredoxin domain [YP_009043724.1]	100% | 97.14%	4e-69
		gp43	Endonuclease	Nucleotide	Endonuclease [YP_009043721.1]	100% | 97.03%	2e-67
		gp50	Ribonucleoside diphosphate reductase^*^	Nucleotide/glutathione	Ribonucleotide reductase [YP_009043714.1]	100% | 99.61%	0
R4C		gp17	Ribonuclease III	Nucleotide	Chain S, ribonuclease III [8GTC_S]	100% | 100.00%	3e-153
		gp30	Nuclease	Nucleotide	Thermonuclease family protein [WP_227271214.1]	98% | 50.00%	3e-28
		gp37	DNA methyltransferase^*^	Nucleotide	DNA methyltransferase [WP_282129374.1]	198% | 71.02%	0
R26L	Trp	gp19	phoH, phosphate starvation-inducible protein^*^	Phosphate metabolism	PhoH family protein [HEY7823769.1]	98% | 66.38%	7e-109
		gp44	Acyl homoserine lactone synthase^*^	Cysteine and methionine metabolism/quorum sensing	Acyl-homoserine-lactone synthase [MEO0342970.1]	92% | 42.68%	2e-23
		gp76	3′ Exoribonuclease, RNase T-like	Nucleotide	3′–5′ Exonuclease [HEY7823714.1]	98% | 55.61%	2e-63
		gp88	dCMP deaminase^*^	Nucleotide	Deoxycytidylate deaminase [YP_009600224.1]	96% | 86.01%	1e-90
		gp90	queE, 7-carboxy-7-deazaguanine synthase^*^	Queuosine biosynthesis	7-Carboxy-7-deazaguanine synthase QueE [HEY7824528.1]	100% | 64.86%	8e-129
		gp95	Exonuclease V	Nucleotide	3′–5′ Exonuclease [HEY7823714.1]	98% | 55.61%	2e-63
		gp98	queF, 7-cyano-7-deazaguanine reductase^*^	Queuosine biosynthesis	NADPH-dependent 7-cyano-7-deazaguanine reductase [UXO93768.1]	99% | 87.18%	3e-96
		gp99	folE, GTP cyclohydrolase IA^*^	Queuosine biosynthesis	GTP cyclohydrolase I FolE [HEY7823432.1]	100% | 78.65%	1e-108
		gp100	queC, 7-cyano-7-deazaguanine synthase^*^	Queuosine biosynthesis	queC-like queuosine biosynthesis [YP_009600206.1]	98% | 85.45%	6e-174
		gp102	queD, 6-pyruvoyl tetrahydropterin synthase^*^	Queuosine biosynthesis	QueD-like 6-pyruvoyl tetrahydropterin [YP_009600203.1]	99% | 84.93%	2e-88

^*^Predicted AMGs are indicated with asterisks.

**Figure 6:**
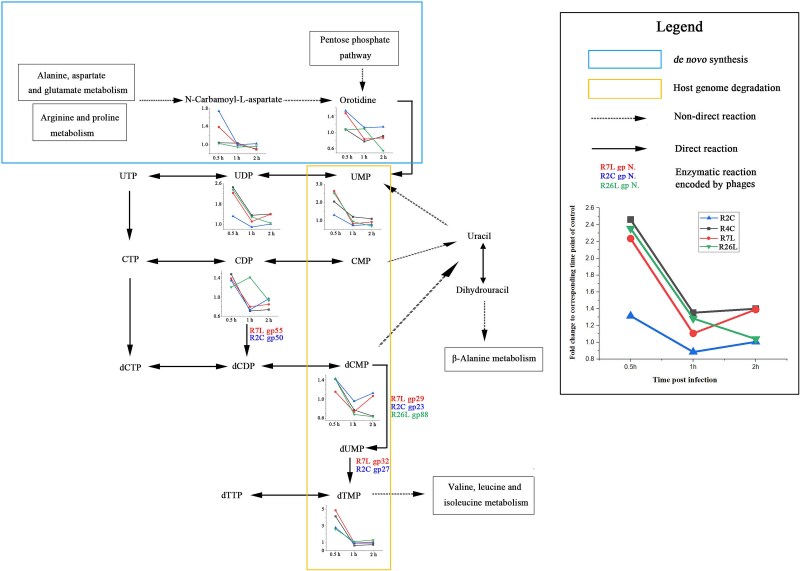
Distinct pyrimidine metabolism strategies of *Dinoroseobacter shibae* phages. Selected metabolites in the pyrimidine metabolism pathway (adapted from KEGG pathway pyrimidine metabolism map00240) are shown to illustrate different pyrimidine metabolism strategies used by phages R2C, R4C, R7L, and R26L. Fold changes of detected metabolites at different stages of infection are presented. The metabolites participating in recycling and *de novo* synthesis of pyrimidine nucleotides are highlighted by boxes. The enzymatic reactions encoded by phages are indicated by phage-encoded AMGs.

All four roseophage genomes contained hypothetical nuclease genes, which may participate in the degradation of host genome [57]. The genomes of R2C and R7L contained abundant AMGs for the *de novo* synthesis of pyrimidine nucleotides, whereas no relevant AMGs were identified in the genomes of R4C and R26L, except for a single AMG (hypothetical dCMP deaminase, gp88) encoded by R26L. This difference suggested that R2C and R7L may adopt different strategies from those of R4C and R26L for expanding the pyrimidine nucleotide pool. Indeed, in the early infection of R4C and R26L, nucleotide monophosphates (UMP, dCMP, and dTMP) in pyrimidine metabolism were significantly increased, whereas the intermediates of *de novo* pyrimidine synthesis (*N*-carbamoyl-l-aspartate and orotidine) remained stable, suggesting that R4C and R26L likely recycled pyrimidine nucleotides by host genome degradation (Fig. 6). In contrast, substantial increases in pyrimidine nucleotide monophosphates as well as intermediates of *de novo* pyrimidine synthesis were recorded for R2C and R7L during early infection. Most likely, the increase in pyrimidine nucleotide monophosphates was caused by the combined effects of recycling and *de novo* synthesis of pyrimidine nucleotides, given that genes involved in both strategies are encoded by the R2C and R7L genomes. The difference in pyrimidine metabolism strategy might be related to the different extent of variation in GC content between phage and host genomes. R26L (62.6%) and R4C (66.8%) had similar genome GC contents to that of the host (66.0%); thus, the recycled nucleotides from the host genome are ready to be incorporated into phage DNA. In contrast, R2C (49.2%) and R7L (49.0%) had much lower genome GC contents than that of the host, necessitating the conversion of cytidine to thymidine. In this context, both R2C and R7L encode relevant AMGs (gp29 and gp32 in R7L, and gp23 and gp27 in R2C) predicted to catalyze the conversion of dCMP to dTMP (Fig. 6). This correlation is further supported by the presence of an AMG encoding hypothetical nucleoside 2-deoxyribosyltransferase (gp4) in the R7L genome, which catalyzes the conversation between different nucleosides (Table 1).

Several other AMGs were identified in the genome of R26L, including genes encoding hypothetical acyl homoserine lactone (AHL) synthase (gp44). AHLs are a class of signaling molecules involved in bacterial quorum sensing, a means of communication between bacteria, enabling behaviors based on population density [58]. A previous report showed that *D. shibae* DFL12 produces three different groups of AHLs, i.e., a C8-homoserine lactone (C8-HSL) and two AHLs with a side chain of 18 carbon atoms, C18-en-HSL and C18-dien-HSL [59]. However, none of these AHLs were identified in this study, possibly due to their extracellular secretory nature. Patzelt *et al*. showed that quorum sensing mediated by AHLs induces phenotypic individualization of *D. shibae* cells rather than population coordination [60]. Thus, the hypothetical AHL synthase in R26L may facilitate the production of AHL and induce morphological heterogeneity of *D. shibae* cells, which might be beneficial in unpredictable environments (e.g., during phytoplankton blooms where grazing and limited resources exert fluctuating selective pressures) [60]. Although some phage-specific metabolic responses can be potentially linked to the predicted AMGs, many other processes cannot be attributed to predicted AMGs, most likely owing to the high number of phage genes that lack functional annotation [61]. It should be noted that the changes in host metabolism induced by phage infection are not only determined by the phage gene repertoire but also by the host metabolic state and dynamic environmental factors [4]. Besides, it should be also noted that the aforementioned potential linkages of phage-elicited alteration in host metabolism with predicted AMGs are just inferred from metabolomic data, and future experimental studies are needed to provide direct evidence for these linkages.

### Potential impact of phage infection on the symbiosis of *D. shibae* and microalgae

**Figure 7:**
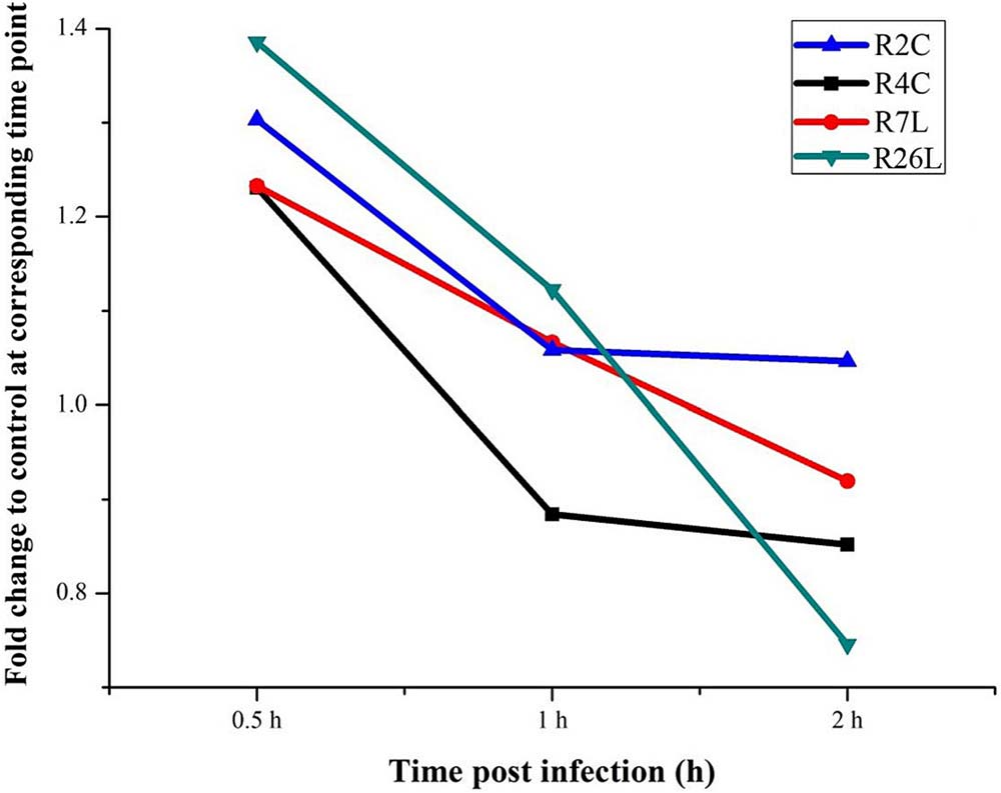
The fold change of vitamin B_1_ (thiamine) content in phage-infected cells to that of control populations.

*Roseobacter* species are often associated with marine microalgae and reach high abundance during phytoplankton blooms [62, 63]. A physical association between *D. shibae* DFL12 and microalgae was suggested by the fact that *D. shibae* DFL12 was isolated from single-washed cells of *P. lima* [19] and was further confirmed by CARD-FISH observations in which many bacteria adhere to the surface of *P. lima* in coculture. *Dinoroseobacter shibae* DFL12 and its closely related strains were also detected from *Alexandrium ostenfeldii* (a toxic dinoflagellate), *Protoceratium reticulatum* (a toxic dinoflagellate isolated from the North Sea), and *Isochrysis galbana* (a nontoxic microalga) [21]. In the *D. shibae*–microalgae symbiosis, *D. shibae* is able to provide the growth-limiting vitamins B_1_ (thiamine) and B_12_ (cobalamin) to its microalgal host, and the microalga is, in turn, able to satisfy the reciprocal vitamin requirements of *D. shibae* [21, 42]. In this study, phage infection had a nonnegligible effect on the synthesis of vitamin B_1_ in the host in an infection stage–dependent manner. As shown in Fig. 7, the content of vitamin B_1_ increased by up to 1.38-fold in the early infection stage for all roseophages. In contrast, the vitamin B_1_ content was stable or decreased for most roseophages in the late infection stage. Therefore, our results emphasize the need to account for phage infections when investigating *Roseobacter*–microalgae interactions and relevant biogeochemical impacts. Metabolites involved in vitamin B_12_ synthesis were not identified; this could be attributed to the strict criterion adopted for accurate metabolite identification in this study.

## Conclusions

We systemically explored global metabolic alterations in *D. shibae* DFL12 in response to infections with four distinct lytic roseophages. The results showed that infected cells are metabolically and functionally different entities from their noninfected counterparts, consistent with the concept of the virocell [3]. Although the central carbon pathway and DNA, amino acid, and coenzyme metabolism were commonly targeted by roseophages, different roseophages elicited distinct host responses. The great diversity in genetic features among roseophages may be the major driver for this metabolic diversity. Furthermore, clear infection stage-specific host responses were observed, corresponding to the different metabolic demands of phage replication in early and late infection stages. Finally, this study provides detailed insight into the potential linkage of phage metabolic effects and AMGs. Each specific set of AMGs carried by phages likely resulted in a unique metabolic phenotype after infection, as suggested by the potential effects of AMGs on the strategies by which phages expand the host pyrimidine pool. With the application of metagenomics and viromics, the discovery and characterization of phage-encoded AMGs are expected to continue, which will likely expand our knowledge of the roles of AMGs in host–virus interactions and relevant biogeochemical cycles.

## Supplementary Material

Supplemental_Figures_ycaf047

Table_S1_new_ycaf047

Table_S2_ycaf047

Table_S3_ycaf047

Table_S4_ycaf047

Table_S5_ycaf047

Table_S6_ycaf047

Table_S7_ycaf047

## Data Availability

The raw matrix data generated from LC–MS and process data during this study are deposited in the Figshare repository (https://doi.org/10.6084/m9.figshare.27215451).

## References

[1] Suttle CA . Marine viruses—major players in the global ecosystem. Nat Rev Microbiol 2007;5:801–12. 10.1038/nrmicro1750 17853907

[2] Rohwer F, Prangishvili D, Lindell D. Roles of viruses in the environment. *Environ Microbiol* 2009;11:2771–4. 10.1111/j.1462-2920.2009.02101.x 19878268

[3] Forterre P . The virocell concept and environmental microbiology. *ISME J* 2013;7:233–6. 10.1038/ismej.2012.110 23038175 PMC3554396

[4] Rosenwasser S, Ziv C, Van Creveld SG et al. Virocell metabolism: metabolic innovations during host–virus interactions in the ocean. *Trends Microbiol* 2016;24:821–32. 10.1016/j.tim.2016.06.006 27395772

[5] Zimmerman AE, Howard-Varona C, Needham DM et al. Metabolic and biogeochemical consequences of viral infection in aquatic ecosystems. *Nat Rev Microbiol* 2020;18:21–34. 10.1038/s41579-019-0270-x 31690825

[6] Breitbart M, Bonnain C, Malki K et al. Phage puppet masters of the marine microbial realm. *Nat Microbiol* 2018;3:754–66. 10.1038/s41564-018-0166-y 29867096

[7] Hurwitz BL, U’Ren JM. Viral metabolic reprogramming in marine ecosystems. *Curr Opin Microbiol* 2016;31:161–8. 10.1016/j.mib.2016.04.002 27088500

[8] Roux S, Brum JR, Dutilh BE et al. Ecogenomics and potential biogeochemical impacts of globally abundant ocean viruses. *Nature* 2016;537:689–93. 10.1038/nature19366 27654921

[9] Jin M, Guo X, Zhang R et al. Diversities and potential biogeochemical impacts of mangrove soil viruses. *Microbiome* 2019;7:1–15. 10.1186/s40168-019-0675-9 30975205 PMC6460857

[10] Ankrah NYD, May AL, Middleton JL et al. Phage infection of an environmentally relevant marine bacterium alters host metabolism and lysate composition. *ISME J* 2014;8:1089–100. 10.1038/ismej.2013.216 24304672 PMC3996693

[11] Jin M, Xu C, Zhang X. The effect of tryptophol on the bacteriophage infection in high-temperature environment. *Appl Microbiol Biotechnol* 2015;99:8101–11. 10.1007/s00253-015-6674-2 25994257

[12] Zhao X, Shen M, Jiang X et al. Transcriptomic and metabolomic profiling of phage–host interactions between PHAGE PaP1 and *Pseudomonas aeruginosa*. *Front Microbiol* 2017;8:548. 10.3389/fmicb.2017.00548 28421049 PMC5377924

[13] De Smet J, Zimmermann M, Kogadeeva M et al. High coverage metabolomic analysis reveals phage-specific alterations to *Pseudomonas aeruginosa* physiology during infection. *ISME J* 2016;10:1823–35. 10.1038/ismej.2016.3 26882266 PMC5029163

[14] Wagner-Döbler I, Biebl H. Environmental biology of the marine *Roseobacter* lineage. *Ann Rev Microbiol* 2006;60:255–80. 10.1146/annurev.micro.60.080805.142115 16719716

[15] Cunliffe M . Correlating carbon monoxide oxidation with cox genes in the abundant marine *Roseobacter* clade. *ISME J* 2011;5:685–91. 10.1038/ismej.2010.170 21068776 PMC3105738

[16] Brinkhoff T, Giebel H-A, Simon M. Diversity, ecology, and genomics of the *Roseobacter* clade: a short overview. *Arch Microbiol* 2008;189:531–9. 10.1007/s00203-008-0353-y 18253713

[17] Martens T, Gram L, Grossart H-P et al. Bacteria of the *Roseobacter* clade show potential for secondary metabolite production. *Microb Ecol* 2007;54:31–42. 10.1007/s00248-006-9165-2 17351813

[18] Buchan A, Collier LS, Neidle EL et al. Key aromatic-ring-cleaving enzyme, protocatechuate 3, 4-dioxygenase, in the ecologically important marine *Roseobacter* lineage. *Appl Environ Microbiol* 2000;66:4662–72. 10.1128/AEM.66.11.4662-4672.2000 11055908 PMC92364

[19] Biebl H, Allgaier M, Tindall BJ et al. *Dinoroseobacter shibae* gen. nov., sp. nov., a new aerobic phototrophic bacterium isolated from dinoflagellates. *Int J Syst Evol Microbiol* 2005;55:1089–96. 10.1099/ijs.0.63511-0 15879238

[20] Allgaier M, Uphoff H, Felske A et al. Aerobic anoxygenic photosynthesis in *Roseobacter* clade bacteria from diverse marine habitats. *Appl Environ Microbiol* 2003;69:5051–9. 10.1128/AEM.69.9.5051-5059.2003 12957886 PMC194994

[21] Wagner-Döbler I, Ballhausen B, Berger M et al. The complete genome sequence of the algal symbiont *Dinoroseobacter shibae*: a hitchhiker’s guide to life in the sea. *ISME J* 2010;4:61–77. 10.1038/ismej.2009.94 19741735

[22] Cai L, Ma R, Chen H et al. A newly isolated roseophage represents a distinct member of *Siphoviridae* family. *Virol J* 2019;16:1–9. 10.1186/s12985-019-1241-6 31694663 PMC6836515

[23] Huang X, Jiao N, Zhang R. The genomic content and context of auxiliary metabolic genes in roseophages. *Environ Microbiol* 2021;23:3743–57. 10.1111/1462-2920.15412 33511765

[24] Labonté JM, Swan BK, Poulos B et al. Single-cell genomics-based analysis of virus–host interactions in marine surface bacterioplankton. *ISME J* 2015;9:2386–99. 10.1038/ismej.2015.48 25848873 PMC4611503

[25] Zhang Y, Zhang F, Yang J et al. Host responses of a marine bacterium, *Roseobacter denitrificans* OCh114, to phage infection. *Arch Microbiol* 2012;194:323–30. 10.1007/s00203-011-0765-y 22033766

[26] Yang Y, Cai L, Wang Y et al. Microarray analysis of gene expression of *Dinoroseobacter shibae* DFL12^T^ in response to phage R2C infection. *Mar Genomics* 2018;42:53–7. 10.1016/j.margen.2018.06.001

[27] Huang X, Yu C, Lu L. Isolation and characterization of a roseophage representing a novel genus in the N4-like *Rhodovirinae* subfamily distributed in estuarine waters. BMC Genomics 2025;26:295. 10.1186/s12864-025-11463-7 PMC11934525 40133813

[28] Cai L, Yang Y, Jiao N et al. Complete genome sequence of vB_DshP-R2C, a N4-like lytic roseophage. *Mar Genomics* 2015;22:15–7. 10.1016/j.margen.2015.03.005 25795023

[29] Wei N, Lu L, Li Y et al. A novel roseosiphovirus infecting *Dinoroseobacter shibae* DFL12^T^ represents a new genus. *BMC Genomics* 2025;26:121. 10.1186/s12864-025-11274-w 39923004 PMC11806900

[30] Bin JH, Bolduc B, Zablocki O et al. Taxonomic assignment of uncultivated prokaryotic virus genomes is enabled by gene-sharing networks. *Nat Biotechnol* 2019;37:632–9. 10.1038/s41587-019-0100-8 31061483

[31] Shannon P, Markiel A, Ozier O et al. Cytoscape: a software environment for integrated models of biomolecular interaction networks. *Genome Res* 2003;13:2498–504. 10.1101/gr.1239303 14597658 PMC403769

[32] Kieft K, Zhou Z, Anantharaman K. VIBRANT: automated recovery, annotation and curation of microbial viruses, and evaluation of viral community function from genomic sequences. *Microbiome* 2020;8:1–23. 10.1186/s40168-020-00867-0 32522236 PMC7288430

[33] Holman JD, Tabb DL, Mallick P. Employing ProteoWizard to convert raw mass spectrometry data. *Curr Protoc Bioinformatics* 2014;46:1–13. 10.1002/0471250953.bi1324s46 PMC4113728 24939128

[34] Smith CA, Want EJ, O'Maille G et al. XCMS: processing mass spectrometry data for metabolite profiling using nonlinear peak alignment, matching, and identification. *Anal Chem* 2006;78:779–87. 10.1021/ac051437y 16448051

[35] Trygg J, Wold S. Orthogonal projections to latent structures (O-PLS). *J Chemom* 2002;16:119–28. 10.1002/cem.695

[36] Kolde R, Kolde MR. Package ‘pheatmap’. R package 2015;1:790.

[37] Pang Z, Chong J, Zhou G et al. MetaboAnalyst 5.0: narrowing the gap between raw spectra and functional insights. *Nucleic Acids Res* 2021;49:W388–96. 10.1093/nar/gkab382 34019663 PMC8265181

[38] Kanehisa M, Goto S. KEGG: Kyoto Encyclopedia of Genes and Genomes. *Nucleic Acids Res* 2000;28:27–30. 10.1093/nar/28.1.27 10592173 PMC102409

[39] Beier N, Kucklick M, Fuchs S et al. Adaptation of *Dinoroseobacter shibae* to oxidative stress and the specific role of RirA. *PLoS One* 2021;16:e0248865. 10.1371/journal.pone.0248865 33780465 PMC8007024

[40] Tomasch J, Gohl R, Bunk B et al. Transcriptional response of the photoheterotrophic marine bacterium *Dinoroseobacter shibae* to changing light regimes. *ISME J* 2021;5:1957–68. 10.1038/ismej.2011.68 PMC3223308 21654848

[41] Neumann A, Patzelt D, Wagner-Döbler I et al. Identification of new N-acylhomoserine lactone signalling compounds of *Dinoroseobacter shibae* DFL12^T^ by overexpression of luxI genes. *Chembiochem* 2013;14:2355–61. 10.1002/cbic.201300424 24218333

[42] Cooper MB, Kazamia E, Helliwell KE et al. Cross-exchange of B-vitamins underpins a mutualistic interaction between *Ostreococcus tauri* and *Dinoroseobacter shibae*. *ISME J* 2019;13:334–45. 10.1038/s41396-018-0274-y 30228381 PMC6331578

[43] Wang H, Tomasch J, Michael V et al. Identification of genetic modules mediating the Jekyll and Hyde interaction of *Dinoroseobacter shibae* with the dinoflagellate *Prorocentrum minimum*. *Front Microbiol* 2015;6:1262. 10.3389/fmicb.2015.01262 26617596 PMC4643747

[44] Zhan Y, Chen F. Bacteriophages that infect marine roseobacters: genomics and ecology. *Environ Microbiol* 2019;21:1885–95. 10.1111/1462-2920.14504 30556267

[45] Yang Y, Cai L, Ma R et al. A novel roseosiphophage isolated from the oligotrophic South China Sea. *Viruses* 2017;9:109. 10.3390/v9050109 28505134 PMC5454422

[46] Chan JZ-M, Millard AD, Mann NH et al. Comparative genomics defines the core genome of the growing N4-like phage genus and identifies N4-like Roseophage specific genes. *Front Microbiol* 2014;5:506. 10.3389/fmicb.2014.00506 25346726 PMC4193335

[47] Nordström A, Want E, Northen T et al. Multiple ionization mass spectrometry strategy used to reveal the complexity of metabolomic. *Anal Chem* 2008;80:421–9. 10.1021/ac701982e 18085752

[48] Howard-Varona C, Lindback MM, Bastien GE et al. Phage-specific metabolic reprogramming of virocells. *ISME J* 2020;14:881–95. 10.1038/s41396-019-0580-z 31896786 PMC7082346

[49] Doron S, Fedida A, Hernández-Prieto MA et al. Transcriptome dynamics of a broad host-range cyanophage and its hosts. *ISME J* 2016;10:1437–55. 10.1038/ismej.2015.210 26623542 PMC5029184

[50] Chandel NS . Glycolysis, vol 13. New York: Cold Spring Harbor 2021. 10.1101/cshperspect.a040576, Lipid Metabolism. PMC8411952 34470787

[51] Goodwin CM, Xu S, Munger J. Stealing the keys to the kitchen: viral manipulation of the host cell metabolic network. *Trends Microbiol* 2015;23:789–98. 10.1016/j.tim.2015.08.007 26439298 PMC4679435

[52] Mittenhuber G . Phylogenetic analyses and comparative genomics of vitamin B6 (pyridoxine) and pyridoxal phosphate biosynthesis pathways. *J Mol Microbiol Biotechnol* 2001;3:1–20. 11200221

[53] Weitz JS, Stock CA, Wilhelm SW et al. A multitrophic model to quantify the effects of marine viruses on microbial food webs and ecosystem processes. *ISME J* 2015;9:1352–64. 10.1038/ismej.2014.220 25635642 PMC4438322

[54] Talmy D, Beckett SJ, Taniguchi DA et al. An empirical model of carbon flow through marine viruses and microzooplankton grazers. *Environ Microbiol* 2019;21:2171–81. 10.1111/1462-2920.14626 30969467

[55] Li B, Du K, Gu S et al. Epigenetic DNA modification N 6-methyladenine inhibits DNA replication by DNA polymerase of *Pseudomonas aeruginosa* phage PaP1. *Chem Res Toxicol* 2019;32:840–9. 10.1021/acs.chemrestox.8b00348 30938985

[56] Enav H, Mandel-Gutfreund Y, Béjà O. Comparative metagenomic analyses reveal viral-induced shifts of host metabolism towards nucleotide biosynthesis. *Microbiome* 2014;2:1–12. 10.1186/2049-2618-2-9 24666644 PMC4022391

[57] Lavigne R, Lecoutere E, Wagemans J et al. A multifaceted study of *Pseudomonas aeruginosa* shutdown by virulent podovirus LUZ19. *MBio* 2013;4:10–1128. 10.1128/mBio.00061-13 PMC3604761 23512961

[58] Fuqua C, Greenberg EP. Listening in on bacteria: acyl-homoserine lactone signalling. *Nat Rev Mol Cell Biol* 2002;3:685–95. 10.1038/nrm907 12209128

[59] Wagner-Döbler I, Thiel V, Eberl L et al. Discovery of complex mixtures of novel long-chain quorum sensing signals in free-living and host-associated marine alphaproteobacteria. *Chembiochem* 2005;6:2195–206. 10.1002/cbic.200500189 16283687

[60] Patzelt D, Wang H, Buchholz I et al. You are what you talk: quorum sensing induces individual morphologies and cell division modes in *Dinoroseobacter shibae*. *ISME J* 2013;7:2274–86. 10.1038/ismej.2013.107 23823498 PMC3834844

[61] Roux S, Hallam SJ, Woyke T et al. Viral dark matter and virus–host interactions resolved from publicly available microbial genomes. *elife* 2015;4:e08490. 10.7554/eLife.08490 26200428 PMC4533152

[62] Bruhn JB, Nielsen KF, Hjelm M et al. Ecology, inhibitory activity, and morphogenesis of a marine antagonistic bacterium belonging to the *Roseobacter* clade. *Appl Environ Microbiol* 2005;71:7263–70. 10.1128/AEM.71.11.7263-7270.2005 16269767 PMC1287604

[63] Buchan A, LeCleir GR, Gulvik CA et al. Master recyclers: features and functions of bacteria associated with phytoplankton blooms. *Nat Rev Microbiol* 2014;12:686–98. 10.1038/nrmicro3326 25134618

