# Supplementary material for: Combined metabolomic and genomic analyses reveal phage-specific and infection stage-specific alterations to marine Roseobacter metabolism
Source: ISME Commun. 2025 Mar 18;5(1):ycaf047. doi: 10.1093/ismeco/ycaf047 (PMC11981692; doi:10.1093/ismeco/ycaf047)
Supplement: Supplemental_Figures_ycaf047 [file supplemental_figures_ycaf047.pdf]

Supplemental Figures

Combined metabolomic and genomic analyses reveal phage-specific and infection stage-specific alterations to marine *Roseobacter* metabolism

Min Jin[1*†], Lanlan Cai[2†], Longfei Lu[3], Meishun Yu[1], Rui Zhang[4*]

[1]State Key Laboratory Breeding Base of Marine Genetic Resource and Laboratory for Southern Marine Science and Engineering Guangdong Laboratory (Zhuhai), Third Institute of Oceanography, Ministry of Natural Resources, Xiamen, 361000, China

[2]Earth, Ocean and Atmospheric Sciences Thrust, The Hong Kong University of Science and Technology (Guangzhou), Guangzhou, China

[3]Fourth Institute of Oceanography, Ministry of Natural Resources, Beihai, Guangxi, China

[4]Archaeal Biology Center, Synthetic Biology Research Center, Shenzhen Key Laboratory of Marine Microbiome Engineering, Key Laboratory of Marine Microbiome Engineering of Guangdong Higher Education Institutes, Institute for Advanced Study, Shenzhen University, Shenzhen 518055, China

† These authors contributed equally to this work.

*Corresponding author (Rui Zhang): E-mail address: ruizhang@szu.edu.cn

*Corresponding author (Min Jin): E-mail address: jinmin@tio.org.cn

a

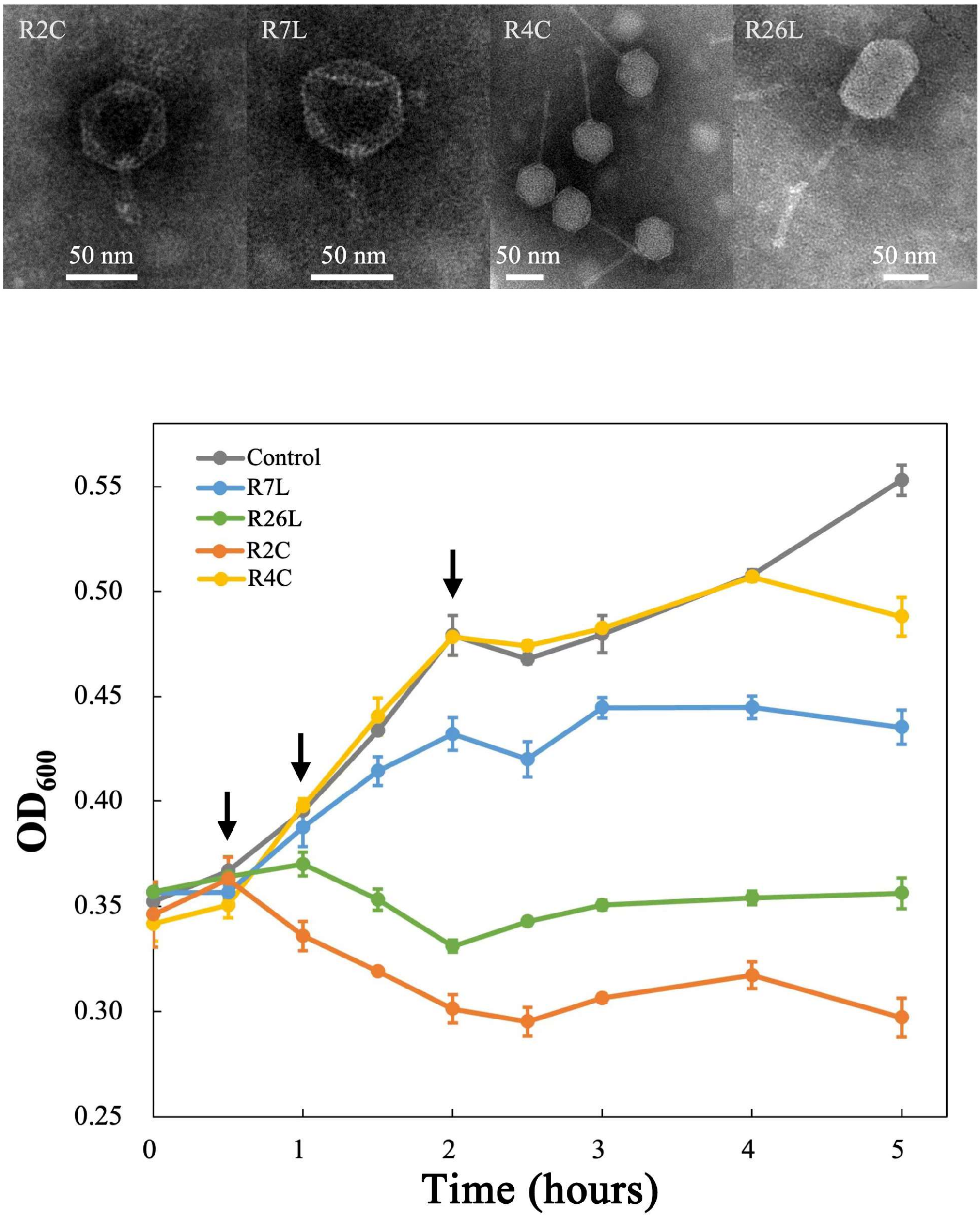

Fig. S1 The morphology and infection phenotypes of four *Dinoroseobacter shibae* phages. (a) The transmission electron microscope images of the four roseophages. **(b)** Killing curve and sampling time points for the four roseophages and the control (without phage infection). Arrows indicate the time points at which host intracellular metabolites were sampled. Average values and standard deviations of three biological replicates are shown.

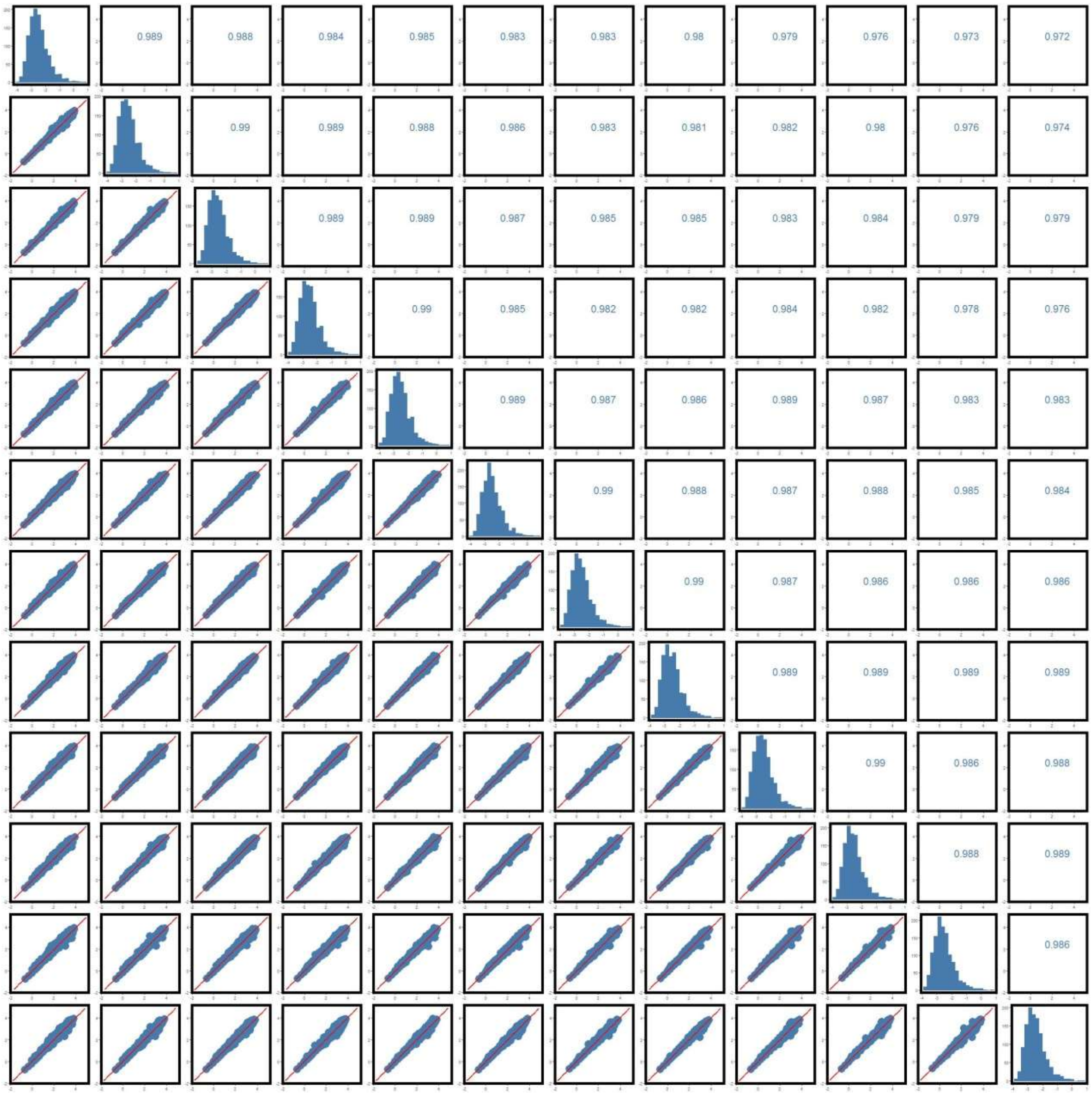

Fig. S2 Correlation analysis of twelve repeated LC-MS/MS analyses on the same QC sample.

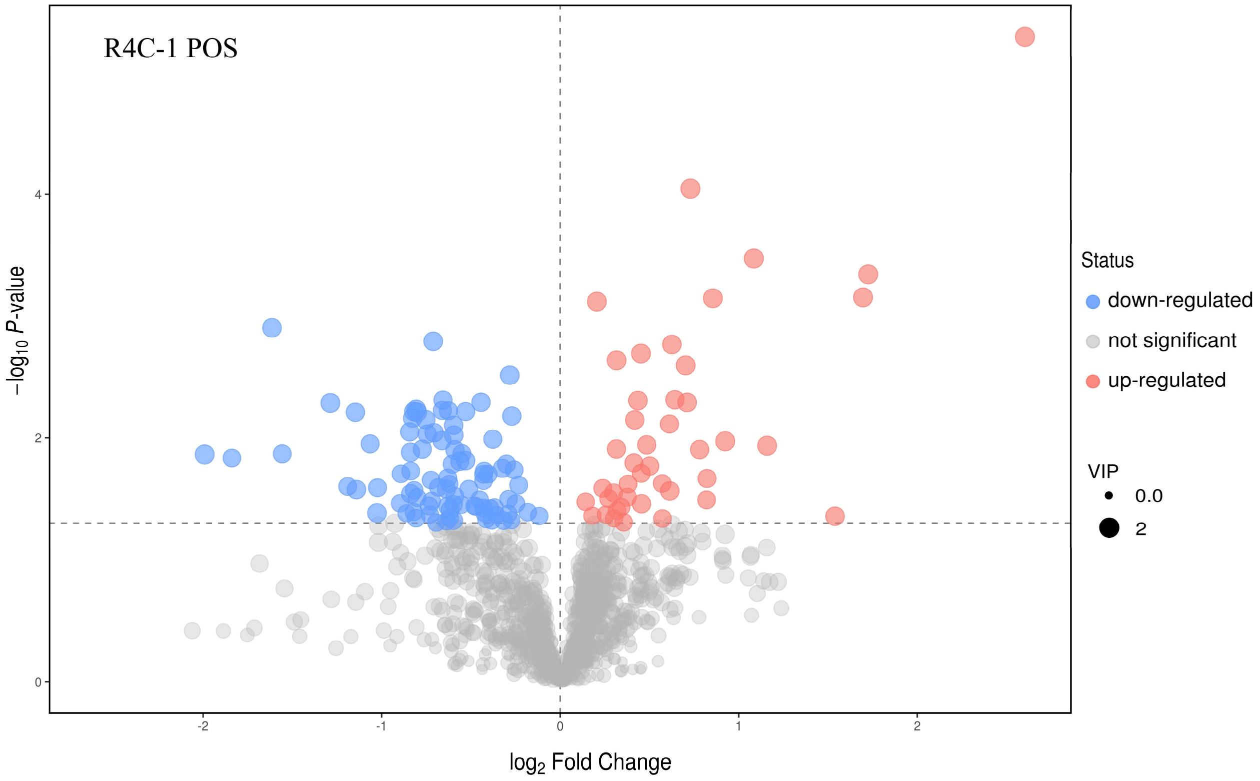

Fig. S3 Volcano plot to identify significantly changed metabolite peaks (*p* ≤ 0.05 and VIP ≥ 1). The volcano plot for R4C-1 at positive mode is shown as an example. The volcano plots for each infection and timepoint are deposited in the Figshare repository (https://doi.org/10.6084/m9.figshare.27215451).

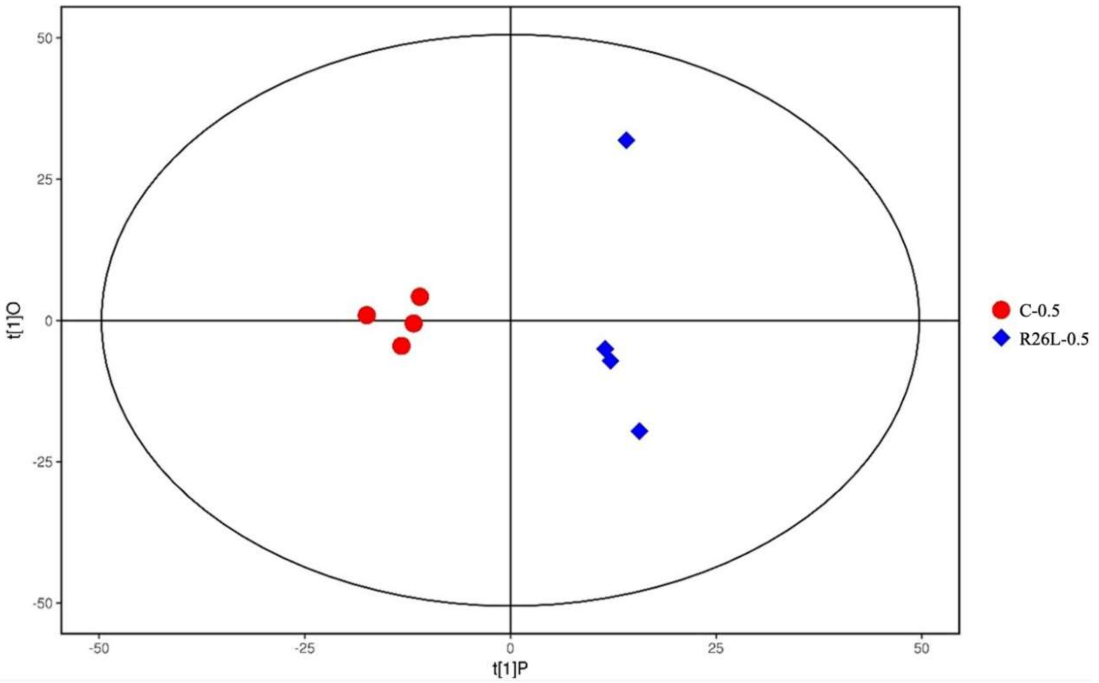

Fig. S4 OPLS-DA analysis between phage-infected host intracellular metabolome and uninfected host intracellular metabolome at the same time point. The OPLS-DA plot for R26C-0.5 at positive mode is shown as an example. The OPLS-DA plots for each infection and timepoint are deposited in the Figshare repository (https://doi.org/10.6084/m9.figshare.27215451).

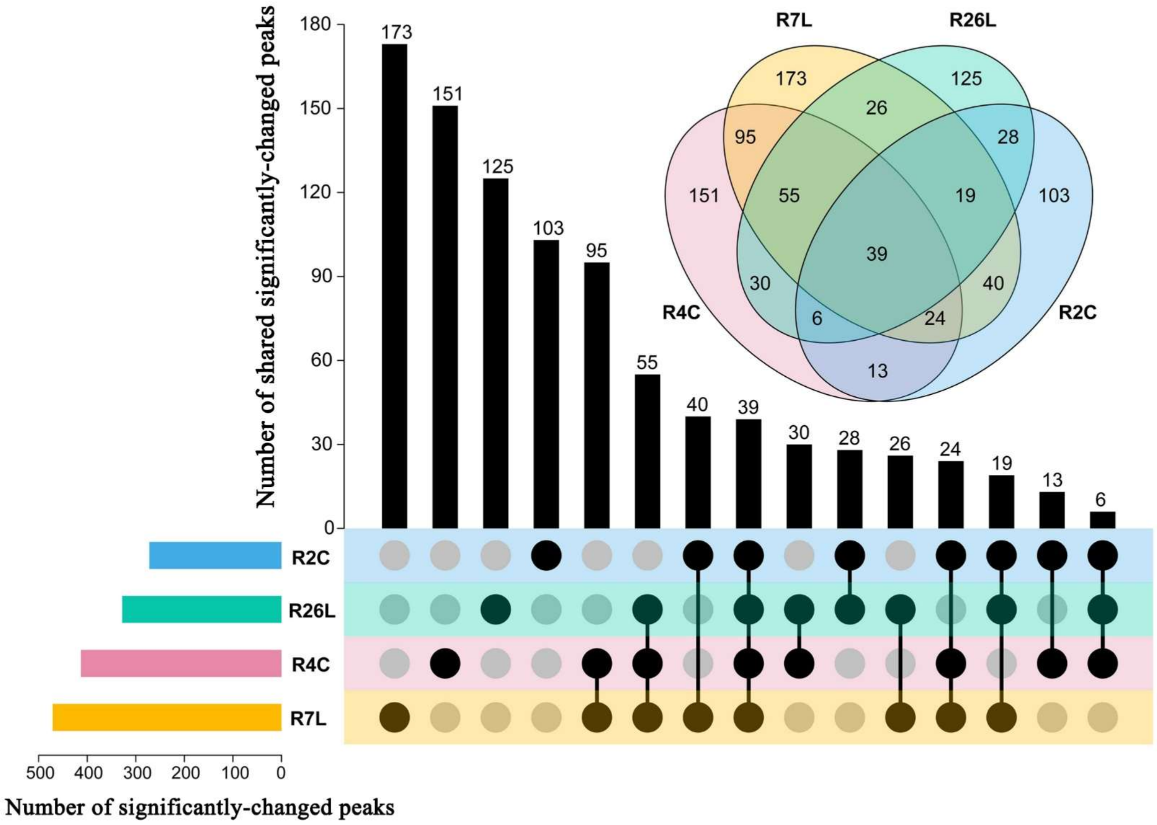

Fig. S5 Venn and upsets plots showing shared significantly changed peaks (*p* ⩽ 0.05 and VIP ⩾ 1 between phage-infected and control populations) among different phage infections.

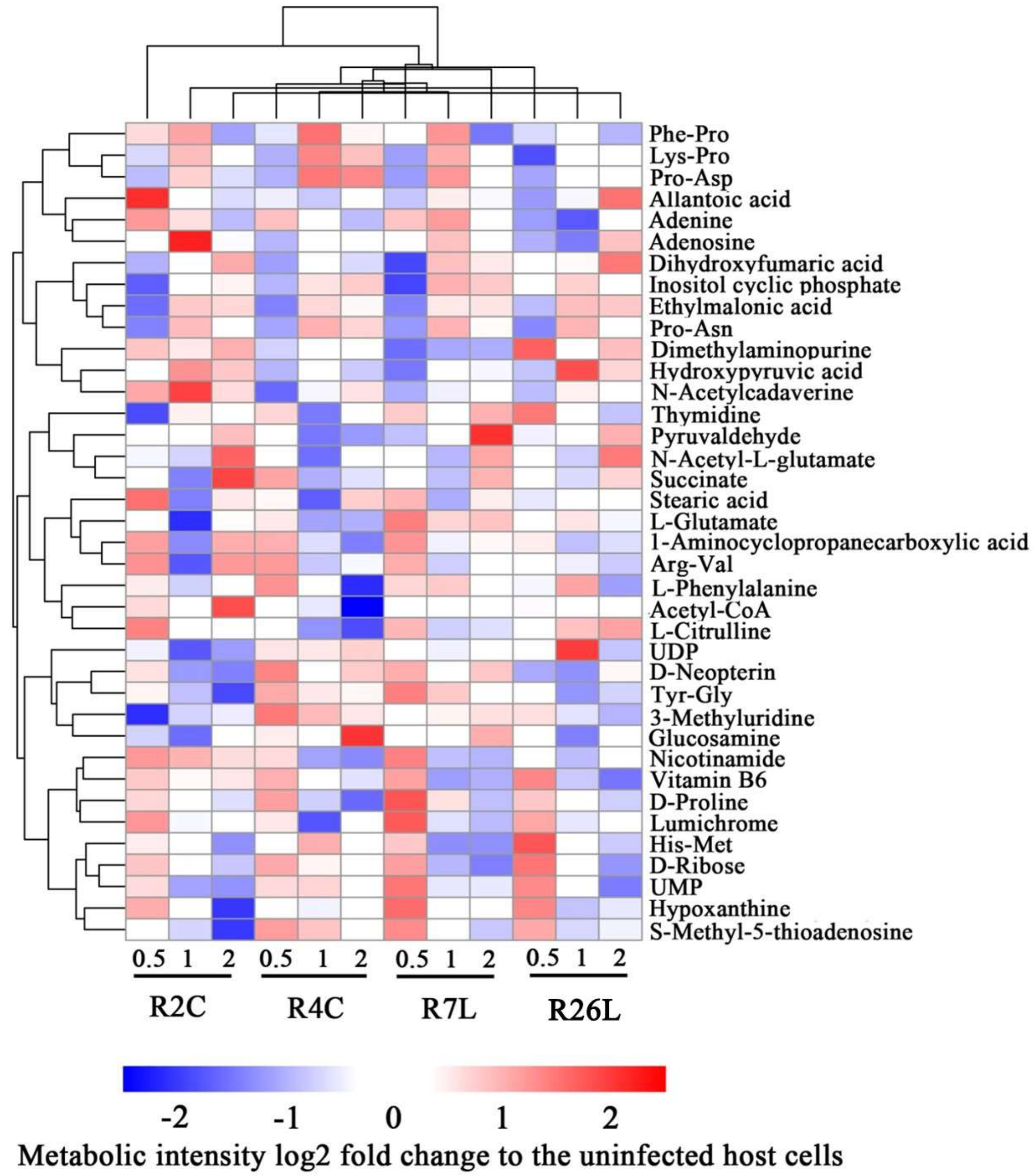

Fig. S6 Hierarchical clustering based on the intensity of significantly changed intracellular metabolites shared in all or three phage infections. The color bar indicates the log2 transformed fold change of metabolite mean intensity of phage-infected cells to that of control populations. Increases in metabolite intensity are shown in red and decreases in blue. The hierarchical trees indicating the relation between different samples and different significantly changed metabolites were shown on the left and top, respectively.
