# Supplementary material for: Combined metabolomic and genomic analyses reveal phage-specific and infection stage-specific alterations to marine Roseobacter metabolism
Source: ISME Commun. 2025 Mar 18;5(1):ycaf047. doi: 10.1093/ismeco/ycaf047 (PMC11981692; doi:10.1093/ismeco/ycaf047)
Supplement: Table_S1_new_ycaf047 [file table_s1_new_ycaf047.docx]

**Table S1 Main characteristics of *Dinoroseobacter shibae* phages used in this study**

| **Phage** | **Family** | **Genus** | **Genome (bp)** | **ORF number** | **GC%** | **Latent period (h)** | **Burst size (viruses per cell)** | **Accession Number** | **Reference** |
| --- | --- | --- | --- | --- | --- | --- | --- | --- | --- |
| vB_DshP-R7L (R7L) | *Schitoviridae* | */* | 75,871 | 85 | 49.0 | 1.0 | 320 | NC_070859.1 | (1) |
| vB_DshP-R2C (R2C) | *Schitoviridae* | *Rhodovirinae* | 74,806 | 85 | 49.2 | 1.5 | 90 | KJ803031.1 | (2) |
| vB_DshS-R4C (R4C) | */* | *Cronusvirus* | 36,291 | 49 | 66.8 | 1.5 | 96 | MK882925 | (3, 4) |
| vB_DshS-R26L (R26L) | */* | */* | 79,535 | 115 | 62.6 | 3.5 | 22 | PP882867 | (5) |

**Reference**

1. Huang X, Yu C, Lu L. Isolation and characterization of a roseophage representing a novel genus in the N4-like *Rhodovirinae* subfamily distributed in estuarine waters. *bioRxiv* 2024 https://doi.org/10.1101/2024.10.08.617335.

2. Cai L, Yang Y, Jiao N, Zhang R. Complete genome sequence of vB_DshP-R2C, a N4-like lytic roseophage. *Marine Genomics* 2015;**22**:15-17.

3. Cai L, Ma R, Chen H, Yang Y, Jiao N, Zhang R. A newly isolated roseophage represents a distinct member of *Siphoviridae* family. *Virology Journal* 2019;**16**:1-9.

4. Huang Y, Sun H, Wei S, Cai L, Liu L, Jiang Y, et al. 2023. Structure and proposed DNA delivery mechanism of a marine roseophage. *Nature Communications* 2023;**14**:3609.

5. Wei N, Lu L, Li Y, Ding B, Cai L, Yang Y. A novel roseosiphovirus infecting *Dinoroseobacter shibae* DFL12^T^ represents a new genus. *BMC Genomics* 2025; **26**(1):1-10.
