# Supplementary material for: Combined metabolomic and genomic analyses reveal phage-specific and infection stage-specific alterations to marine Roseobacter metabolism
Source: ISME Commun. 2025 Mar 18;5(1):ycaf047. doi: 10.1093/ismeco/ycaf047 (PMC11981692; doi:10.1093/ismeco/ycaf047)
Supplement: Table_S6_ycaf047 [file table_s6_ycaf047.docx]

**Table S6** Number and percentage of total peaks significantly changed (*p* ≤ 0.05 and VIP ≥ 1) between phage-infected and control populations during the course of infection of *D. shibae* DFL12 with four different phages.

|  | **R2C** | | **R4C** | | **R7L** | | **R26L** | |
| --- | --- | --- | --- | --- | --- | --- | --- | --- |
|  | Increased | Decreased | Increased | Decreased | Increased | Decreased | Increased | Decreased |
| **0.5 h** | 67（2.91%） | 8（0.35%） | 188（8.17%） | 23（1.00%） | 211（9.17%） | 55（2.39%） | 96（4.17%） | 22（0.96%） |
| **1 h** | 57（2.48%） | 34（1.48%） | 68（2.96%） | 104（4.52%） | 50（2.17%） | 77（3.35%） | 96（4.17%） | 34（1.48%） |
| **2 h** | 125（5.43%） | 30（1.30%） | 72（3.13%） | 53（2.30%） | 108（4.69%） | 77（3.35） | 50（2.17%） | 64（2.78%） |
